# The Influence of Vitamin C on Postoperative Pain in Patients with Trochanteric Femur Fracture

**DOI:** 10.5812/aapm-169833

**Published:** 2026-02-28

**Authors:** Mirza Sivro, Faruk Hodžić, Tarik Branković

**Affiliations:** 1 Department of Orthopedics and Traumatology, Cantonal Hospital Zenica, Zenica, Bosnia and Herzegovina

**Keywords:** Analgesics, Ascorbic Acid, Femoral Fractures, Pain Management

## Abstract

**Background:**

Trochanteric femur fractures constitute nearly half of all hip fractures and represent the most commonly surgically treated fracture type overall. Postoperative pain is linked to increased morbidity and poorer functional recovery, and its management remains challenging due to the adverse effects associated with commonly prescribed analgesics.

**Objectives:**

This study aimed to assess the influence of oral vitamin C supplementation on postoperative pain intensity in patients with trochanteric femur fractures managed with intramedullary nailing.

**Methods:**

A prospective, randomized, single-blinded, placebo-controlled clinical trial was performed involving 70 patients allocated to either a vitamin C group or a control group. Participants in the vitamin C group received 1 g of oral vitamin C daily for 40 postoperative days, whereas the control group received a placebo for the same duration. Baseline demographic and clinical characteristics, Visual Analogue Scale (VAS) pain scores, total postoperative metamizole consumption, Harris Hip Score (HHS) values, and complication rates were recorded and compared between groups.

**Results:**

No statistically significant differences were observed between groups regarding age, gender distribution, length of hospital stay, fracture side, or fracture classification. The cumulative postoperative VAS scores were significantly lower in the vitamin C group compared with the control group (P = 0.0000). Median postoperative metamizole consumption was lower in the vitamin C group, with a significant difference compared with the control group (P = 0.020). Harris Hip Score values assessed at 6 and 12 weeks postoperatively did not differ significantly between groups (P = 0.760 and P = 0.796, respectively).

**Conclusions:**

The findings indicate that vitamin C supplementation significantly reduced subjective postoperative pain and analgesic consumption, suggesting its potential usefulness as an adjunctive measure for pain management following hip fracture surgery.

## 1. Background

Hip fractures are a prevalent health concern among the elderly population and are associated with substantial morbidity, mortality, and extended hospital stays. With increasing life expectancy, the worldwide incidence of hip fractures is expected to reach 6.26 million within the next 25 years (1). Reported mortality rates during the first postoperative year range from 20% to 24% (2). Trochanteric fractures account for approximately half of all hip fractures. Intramedullary nailing is currently the preferred surgical approach because it offers shorter operative time, reduced intraoperative blood loss, and earlier mobilization (3). Despite advancements in fixation techniques, overall mortality rates and functional outcomes have shown minimal improvement over the past 25 years (4).

Postoperative pain following hip fracture surgery is associated with prolonged hospitalization, limited mobility, and impaired functional recovery. Many patients experience persistent pain for months after surgery, and pain following hospital discharge significantly interferes with rehabilitation. This contributes to reduced physical activity, diminished mobility, and increased disability (5). Given that hip fractures predominantly occur in elderly individuals, pain management is particularly complex due to age-related physiological changes and organ dysfunction (6).

Nonsteroidal anti-inflammatory drugs (NSAIDs) and opioids are widely used for postoperative analgesia but are associated with adverse effects such as gastrointestinal bleeding, cardiovascular events, nausea, vomiting, and respiratory depression (7). Vitamin C (ascorbic acid) is a water-soluble vitamin with strong antioxidant properties, capable of neutralizing reactive oxygen species and suppressing proinflammatory cytokines (8). Additionally, it exhibits antinociceptive, neuroprotective, and neuromodulatory effects, influencing glutamate and dopamine signaling via modulation of NMDA receptor redox states (9). Vitamin C is generally safe at doses up to 2 g per day; however, potential adverse effects include gastrointestinal discomfort, diarrhea, hyperoxaluria, and, in acidic urine, renal oxalate stone formation (10).

Previous research has demonstrated that vitamin C supplementation may reduce postoperative pain and prevent complex regional pain syndrome (CRPS) following foot, ankle, and wrist surgeries, enhance tendon healing after rotator cuff repair, and decrease the incidence of arthrofibrosis after total knee arthroplasty (11-13). Nevertheless, the role of vitamin C following hip fracture surgery has received limited attention, with only one study suggesting that postoperative ascorbic acid levels at discharge may influence outcomes in patients with proximal femur fractures (14).

## 2. Objectives

The primary objective of this study was to evaluate the effect of vitamin C supplementation on postoperative pain severity in patients undergoing intramedullary nailing for trochanteric femur fracture.

## 3. Methods

### 3.1. Study Design and Participants

This prospective, randomized, single-blinded, placebo-controlled study included 70 patients who underwent surgical treatment between July 2023 and June 2025 at the Department of Orthopedics and Traumatology, Cantonal Hospital Zenica, Bosnia and Herzegovina. The dosage and duration of oral vitamin C administration were selected based on previous studies involving orthopedic populations (11-13). Sample size calculation, derived from comparable patient cohorts, indicated that 35 patients per group were required to achieve 80% statistical power at an alpha level of 0.05, assuming expected VAS means of 2 for the vitamin C group and 3.6 for the control group, with a standard deviation of 2.4.

The primary outcome measures were postoperative pain severity and analgesic consumption. Secondary outcomes included functional recovery, postoperative medical and surgical complications, and vitamin C-related adverse effects.

Inclusion criteria were: AO-classified trochanteric fractures (types 31.A1, A2, and A3), age ≥ 18 years, injury sustained within two weeks, and written consent to participate. Exclusion criteria included: Previous hip surgery, polytrauma, diabetes mellitus, prior limb amputation, renal insufficiency, cardiorespiratory insufficiency, coagulation disorders, malignancy, pathological fractures, contraindications to surgery, medication allergies, neurological deficits, and refusal to participate.

Each participant was assigned a unique identification number to ensure confidentiality during statistical analysis.

The study was retrospectively registered in the ISRCTN registry with study registration number ISRCTN13266677 on 12 January 2026 (https://www.isrctn.com/ISRCTN13266677).

### 3.2. Study Design and Procedures

After verification of eligibility criteria, patients were randomly allocated to either the vitamin C group or the control group using a computerized random number generator, with 35 patients in each group. Participants were blinded to both group allocation and the administered substance. The vitamin C group received 1 g of oral vitamin C daily, administered as two 500 mg tablets taken in the morning and evening, for 40 postoperative days. The control group received placebo tablets identical in size, color, and shape, taken twice daily for the same duration. Treatment adherence was monitored using medication log sheets during follow-up visits, with acceptable compliance defined as ≥ 80%.

All surgical procedures were performed under general anesthesia. Premedication was performed with intravenous midazolam at a dose of 0.02 - 0.05 mg/kg. Induction of anesthesia was achieved with propofol (1.5 - 2.5 mg/kg IV) in combination with fentanyl (1 - 2 μg/kg IV) for analgesia. Neuromuscular blockade to facilitate endotracheal intubation was achieved with vecuronium bromide (0.08 - 0.1 mg/kg IV). Anesthesia was maintained with sevoflurane inhalation (approximately 1 - 2% end-tidal concentration), adjusted according to clinical parameters and standard intraoperative monitoring. Standard monitoring, including electrocardiography, noninvasive blood pressure measurement, pulse oximetry, and capnography, was applied throughout the procedure. In the recovery room, analgesia was provided intravenously using metamizole (10 - 15 mg/kg) and tramadol (1 - 2 mg/kg) when required for pain control. During postoperative hospitalization, metamizole was used for pain control and administered intravenously in doses of a maximum of 5 g daily. In cases of insufficient pain control or severe pain, tramadol was administered in a total daily dose that did not exceed 400 mg/day.

Osteosynthesis was achieved using a third-generation short cephalomedullary nail in all cases. Prophylactic antibiotics consisted of a first-generation cephalosporin, and thromboprophylaxis was provided with enoxaparin 40 mg administered subcutaneously once daily for 35 postoperative days.

Baseline patient characteristics were recorded for both groups. Surgical drains were removed on the second postoperative day, followed by a control radiograph. Additional radiographic evaluations were performed at 6 and 12 weeks postoperatively. Follow-up assessments occurred at 2, 6, and 12 weeks to document complications and vitamin C-related side effects. Pain intensity was evaluated using a 10-cm Visual Analogue Scale (VAS) (15), ranging from 0 (no pain) to 10 (worst imaginable pain), on the second postoperative day and at 2, 6, and 12 weeks. VAS assessments were conducted by the principal investigator after standardized written instructions were provided to participants. The total dose of postoperative metamizole consumption in milligrams was recorded for each participant.

Functional outcomes were assessed using the Harris Hip Score (HHS) at 6 and 12 weeks postoperatively (16). All patients adhered to the same standardized rehabilitation protocol following hospital discharge.

### 3.3. Ethics

The study protocol was approved by the Cantonal Hospital Zenica Ethical Committee (No 00-03-35-958-10/23), and written informed consent was obtained from all participants. All procedures were carried out in accordance with the ethical standards of medical deontology and the Declaration of Helsinki.

### 3.4. Statistical Analysis

Baseline characteristics were summarized using descriptive statistics. Normally distributed variables were expressed as mean ± standard deviation, while non-normally distributed variables were reported as median with interquartile range. Categorical variables were analyzed using the χ² test or Fisher’s exact test, and continuous variables were compared using Student’s *t*-test or the Mann–Whitney U test, as appropriate. Cumulative pain was quantified by calculating the area under the curve (AUC) from serial VAS measurements. Statistical significance was defined as P < 0.05. Data analysis was performed using SPSS Statistics version 20 (SPSS Inc., Chicago, IL, USA).

## 4. Results

Among the 70 enrolled patients, 20 (28.6%) were male and 50 (71.4%) were female, with no significant difference between groups (P = 0.597). Age distribution was comparable (P = 0.956), as was the duration of hospitalization (P = 0.782). No statistically significant differences were identified regarding fracture side or fracture type (P = 0.473 and P = 0.445, respectively). Of the expected 2800 tablets per group, compliance was 81% in the control group and 82% in the vitamin C group (Table 1). 

**Table 1.** Baseline Characteristics of Patients Between the Groups ^a^

Variables	Control Group	Vitamin C Group	P-Value
**Gender**			0.597
Male	11 (15.7)	9 (12.9)	
Female	24 (34.3)	26 (37.1)	
**Age**	78.46 ± 8.64	78.34 ± 8.61	0.956
**Hospitalization time**	15.71 ±5.65	16.09 ± 5.96	0.782
**Side of fracture**			0.473
Right	18 (25.7)	15 (21.4)	
Left	17 (24.3)	20 (28.6)	
**Type of fracture (AO)**			0.445
A1	9 (12.9)	6 (8.6)	
A2	25 (35.7)	26 (37.1)	
A3	1 (1.4)	3 (4.3)	
**Compliance (%)**	81	82	-
**Tablets; predicted/consumed**	2800/2268	2800/2296	-

Abbreviations: AO, Arbeitsgemeinschaft für Osteosynthesefragen.

^a^ Values are expressed as No. (%) or mean ± SD.

Median VAS scores were consistently lower in the vitamin C group on the second postoperative day and at the second postoperative week, with highly significant differences (P = 0.0000). Significant differences were also observed at 6 and 12 weeks postoperatively (P = 0.002 and P = 0.020, respectively). AUC analysis confirmed significantly lower cumulative pain in the vitamin C group compared with controls (P = 0.0000). Median metamizole consumption during postoperative hospitalization was lower in the control group than in the vitamin C group, with a significant difference (P = 0.020). Median HHS values were marginally higher in the control group at both 6 and 12 weeks; however, these differences were not statistically significant (P = 0.760 and P = 0.796). No adverse effects related to vitamin C administration were reported (Table 2). 

**Table 2.** Primary and Secondary Outcomes Between the Groups ^a^

Outcomes	Control Group	Vitamin C Group	P-Value
**VAS 2. (d)**	6 (5 - 8)	5 (5 - 6)	0.0000
**VAS 2. (wk)**	4 (3 - 5)	3 (2 - 4)	0.0000
**VAS 6. (wk)**	3 (2 - 3)	2 (2 - 2)	0.002
**VAS 12. (wk)**	2 (1 - 2)	1 (1 - 2)	0.020
**VAS (AUC)**	3.13 (2.15 - 3.51)	2.15 (1.85 - 2.46)	0.0000
**Metamizole (mg)**	5000 (2500 - 5000)	3250 (2500 - 4500)	0.020
**HHS 6 (wk)**	57.5 (45.7 - 65.225)	57 (53.25 - 61.85)	0.760
**HHS 12 (wk)**	72.8 (63.45 - 79.5)	71.7 (65.65 - 75.85)	0.796
**Medical complications**			
**UTI**	3	0	0.239
**Delirium**	3	1	0.307
**DVT**	1	0	0.500
**Surgical complications**			
**Wound infection**	1	0	0.307
**Cut-out**	2	2	1.000
**Adverse effects of Vitamin C**	-	0	-

Abbreviations: VAS, Visual Analogue Scale; HHS, Harris Hip Score; AUC, area under curve; UTI, urinary tract infection; DVT, deep vein thrombosis.

^a^ Values are expressed as median and interquartile range (IQR) or No.

Complications included one superficial wound infection in the control group and cephalic screw cut-out in two patients from each group. No urinary tract infections occurred in the vitamin C group, whereas three cases were recorded in the control group (P = 0.239). Postoperative delirium was documented in three control patients, and one control patient developed deep vein thrombosis (Table 2). 

## 5. Discussion

Trochanteric fractures predominantly occur in individuals older than 70 years and are more common in women (17). The median patient age in this study was approximately 80 years, with female predominance, aligning with previous findings (18).

A meta-analysis of seven randomized controlled trials evaluating perioperative vitamin C administration in abdominal and gynecological surgeries demonstrated greater reductions in postoperative pain and opioid consumption within the first 48 hours among patients receiving intravenous vitamin C (19). Similarly, Han et al. reported significantly lower VAS scores at 24 hours postoperatively in patients who received a single intravenous dose of 3 g vitamin C compared with controls (20). In contrast to these studies, the present investigation demonstrated significantly lower VAS scores at 48 hours following oral vitamin C administration. Evaluation on the second postoperative day was particularly relevant, as this coincided with drain removal, postoperative radiographic assessment, and initiation of supervised mobilization. Notably, orthopedic patients were not included in the aforementioned meta-analysis.

Another meta-analysis by Hung et al. examining oral vitamin C supplementation in various orthopedic procedures reported a significantly reduced incidence of CRPS at 12 months, without significant differences in pain intensity or functional outcomes at earlier follow-up intervals (21).

Jain et al. (22) investigated vitamin C supplementation following foot and ankle fracture surgery and reported significantly lower VAS scores at the first, second, and sixth postoperative weeks as well as lower analgesic consumption at the end of the sixth week in patients receiving 1 g daily, findings consistent with the present study.

There were no significant differences in functional outcome between the groups of patients. Functional recovery after hip fracture surgery is multifactorial and influenced by variables such as age, sex, and fracture characteristics (23, 24).

The occurrence of cephalic screw migration in both groups underscores the importance of optimal surgical technique, including appropriate tip-to-apex distance and anatomical reduction prior to nail insertion (25, 26). The absence of urinary tract infections in the vitamin C group supports existing evidence that vitamin C may reduce urinary tract infection risk through urinary acidification (27, 28). Postoperative delirium, a common complication in elderly hip fracture patients, was observed only in the control group, with reported incidences in the literature ranging widely from 4.7% to 74% (29). The low incidence in this study may reflect stringent exclusion criteria and limited sample size. Vitamin C may also enhance wound healing by promoting type I collagen synthesis, potentially lowering postoperative infection risk (30).

A limitation of this study is that only the participants were blinded, while the investigators responsible for outcome assessment were not. The lack of blinding among outcome assessors may have introduced potential bias, particularly in the evaluation of subjective outcomes such as pain. However, to reduce this potential bias and provide a more objective measure of postoperative pain, analgesic consumption was also recorded and analyzed as an additional outcome measure, and pain scores were assessed only by the principal investigator. A larger number of patients and longer follow-up are needed to signify the clinical difference and generalizability of the findings regarding functional outcome and medical complications.

### 5.1. Conclusions

In summary, this study demonstrated a significant reduction in subjective postoperative pain and analgesic consumption among patients receiving vitamin C supplementation. These findings suggest that vitamin C may be considered an effective adjunctive agent for postoperative pain management in patients undergoing surgery for hip fractures.

## Data Availability

The dataset presented in the study is available on request from the corresponding author during submission or after publication.

## Clinical Trial Registration Code:

The study was registered in ISRCTN registry with study registration number ISRCTN13266677 on 12 January 2026 (Link: https://www.isrctn.com/ISRCTN13266677).
